# The Other in Me: Interpersonal Multisensory Stimulation Changes the Mental Representation of the Self

**DOI:** 10.1371/journal.pone.0040682

**Published:** 2012-07-13

**Authors:** Ana Tajadura-Jiménez, Stephanie Grehl, Manos Tsakiris

**Affiliations:** 1 Department of Psychology, Royal Holloway, University of London, Egham, Surrey, United Kingdom; 2 Experimental and Regenerative Neurosciences, School of Animal Biology, University of Western Australia, Western Australia, Australia; French National Centre for Scientific Research, France

## Abstract

**Background:**

Recent studies have shown that the well-known effect of multisensory stimulation on body-awareness can be extended to self-recognition. Seeing someone else’s face being touched at the same time as one’s own face elicits changes in the mental representation of the self-face. We sought to further elucidate the underlying mechanisms and the effects of interpersonal multisensory stimulation (IMS) on the mental representation of the self and others.

**Methodology/Principal Findings:**

Participants saw an unfamiliar face being touched synchronously or asynchronously with their own face, as if they were looking in the mirror. Following synchronous, but not asynchronous, IMS, participants assimilated features of the other’s face in the mental representation of their own face as evidenced by the change in the point of subjective equality for morphed pictures of the two faces. Interestingly, synchronous IMS resulted in a unidirectional change in the self-other distinction, affecting recognition of one’s own face, but not recognition of the other’s face. The participants’ autonomic responses to objects approaching the other’s face were higher following synchronous than asynchronous IMS, but this increase was not specific to the pattern of IMS in interaction with the viewed object. Finally, synchronous, as compared to asynchronous, IMS resulted in significant differences in participants’ ratings of their experience, but unlike other bodily illusions, positive changes in subjective experience were related to the perceived physical similarity between the two faces, and not to identification.

**Conclusions/Significance:**

Synchronous IMS produces quantifiable changes in the mental representations of one’s face, as measured behaviorally. Changes in autonomic responses and in the subjective experience of self-identification were broadly consistent with patterns observed in other bodily illusions, but less robust. Overall, shared multisensory experiences between self and other can change the mental representation of one’s identity, and the perceived similarity of others relative to one’s self.

## Introduction

Our face is the most distinctive feature of our physical appearance, and one of the key ways by which we become known as individuals, both to ourselves and to others. Mirror self-recognition is a key feature of self-awareness and identity [1], [2]. The ability to recognize oneself in a mirror is taken as evidence of a basic form of self-awareness in non-human primates [1], [3] and human infants [4]. This ability for self-face recognition is also claimed to be fundamental to the awareness of being a self among others like us [5], upon which more complex forms of self-identity are built, such as a diachronic sense of self [6], and the expression of social emotions (e.g., embarrassment, pride and guilt [7]). Given the importance of mirror self-recognition for the awareness of an external, “objectified”, dimension of the self, it is unsurprising that recent research has attempted to elucidate the neurocognitive processes engaged in recognizing our own face.

Accumulating evidence favors a right hemispheric specificity in frontoparietal areas responsible for self-face recognition [8]. This is supported by case studies of delusional misidentification syndrome, following right frontoparietal damage, whereby patients misidentify their own face in the mirror [9], and by recent fMRI studies of self-face recognition (for a review see [10]). For example, Uddin et al. [11] reported activations in the right inferior parietal lobule, inferior frontal gyrus and inferior occipital gyrus. These regions were described as a unique network within the “mirror neuron system”, responsible for detecting a match between an external stimulus and the self. Devue et al. [12] reported that visual self-recognition of both faces and bodies activated the right inferior frontal gyrus and the right insula (see also [13], [14]). To further investigate the causal role of these areas in self-other discrimination, Uddin et al. [15] used repetitive transcranial magnetic stimulation (rTMS) over the right inferior parietal lobe (rIPL), which selectively disrupted performance on a self-other discrimination task, whereas rTMS to the left IPL had no effect. Heinisch et al. [16] used rTMS over the prefrontal and the temporoparietal junction (TPJ) prior to measuring face recognition performance with a video morphing task. In one condition, participants saw the face of someone else being morphed into the self-face, and they were asked to stop the movie when the face depicted started to look more like the self-face. In a second condition, participants saw their own face being morphed into the face of someone else, and they were asked to stop the movie when the depicted face started to look more like someone else than like self. rTMS over the right TPJ, but not over the left TPJ, biased self-other discrimination but only in the “other to self” direction of morphing, without any effect on the “self to other” direction of morphing. According to the authors, rTPJ stimulation resulted in a less conservative self-recognition criterion, while recognition of other faces was not affected. While the available neuroimaging data seem to support the hypothesis of the right hemispheric specificity for mirror self-recognition, and allude to a comparison process between an external stimulus and a mental representation of the self, they tell us little in terms of the cognitive processes involved in the acquisition, maintenance and updating of self-face representations.

Behavioral research emphasizes the role of mnemonic representations of one’s face [17]–[21], suggesting that I recognize myself in the mirror because I know the person I see looks like me. In contrast, recent studies that investigated self-recognition of bodily movements across development [22] and the sense of body-ownership [23] emphasize the primary role of multisensory integration for body-awareness, over the role of memory of one’s body appearance. On this view, in the same way that I recognize my body through multisensory integration, I may recognize and form a mental representation of my own face because the mirror reflection moves when I move, and I see it being touched when I feel touch myself. Indeed, the everyday encounter of one’s reflection in the mirror involves a continuous integration of motor, proprioceptive, tactile and visual signals, as every touch on one’s face is mirrored by a compatible visual event. Therefore, mental representations of one’s own face would be constructed and possible updated or altered by multisensory input. Research on body-awareness suggests that multisensory processing can indeed update representations of one’s body, such as the sense of body-ownership [23], and the perceived appearance of one’s body, also known as “body image” [24]. In the rubber hand illusion (RHI), synchronous visuo-tactile stimulation between a rubber hand and one’s own unseen hand generates the feeling that the rubber hand is part of one’s body [25]. Comparable findings have been shown after multisensory stimulation of whole bodies [26]–[29] and in the body-swap illusion [30]. These bodily illusions demonstrate the efficiency of current multisensory input in determining the experience of a minimal 1^st^ person-perspective [28], [29], self-location [27] and self-identification [28]–[30], three critical dimensions of the experience of selfhood [31].

Therefore, accumulating evidence suggests that multisensory integration can be used to produce, or even alter, the sense of self. This hypothesis has been recently extended to self-face representation. Tsakiris [32] showed that synchronous, but not asynchronous, visuo-tactile stimulation between the participant’s own face and a morphed face, containing a blending of the facial features of the participant (50%) with the features of someone else’s face (50%), produced a measurable bias in self-face recognition. A self-recognition task, performed before and after exposure to both synchronous and asynchronous visuo-tactile stimulation, revealed a significant change in the participants’ self-recognition judgments only after exposure to synchronous stimulation; participants accepted as self-stimuli faces that contained a significantly higher percentage of the other’s face. Similar effects were reported in the description of the “enfacement illusion” by Sforza et al. [33], who used exposure to visuo-tactile stimulation delivered to the participant’s face and, unlike Tsakiris [32], the familiar face of a friend or colleague who was physically present. In Sforza et al. [33], the self-recognition task consisted of participants evaluating the amount of self-features in a set of morphed images with different percentages of self and other’s face presented in a random order. Unfortunately, data for a baseline condition, not affected by stroking, was collected during a separate session after the main experiment, and thus this study lacked a critical pre-stimulation behavioral task that would have allowed the *direct* comparison between judgments performed before and after visuo-tactile stimulation. Extending the behavioral results of Tsakiris [32] and Sforza et al. [33], Paladino et al. [34] exposed participants to visuo-tactile stimulation delivered to their face and another unfamiliar face, and showed that synchronous multisensory stimulation might also influence social cognition processes of inference and conformity, and the perceived physical resemblance between one’s own and the other’s face.

While the three studies to date [32]–[34] that have looked into the effect of multisensory stimulation on self-recognition lend support to the hypothesis that multisensory processes influence the mental representation of the self, all three studies had methodological confounds that limit the interpretation of their results. Tsakiris [32] used a self-other morphed face during visuo-tactile stimulation, rather than an unfamiliar other; Sforza et al. [33] used a familiar face during visuo-tactile stimulation and lacked a baseline self-recognition task prior to stimulation; and Paladino et al. [34] lacked a behavioral self-recognition task and did not control for the gender of the viewed model.

We therefore sought, first, to improve the experimental methods used to investigate the *effect* of interpersonal multisensory stimulation (IMS) on the mental representation of one’s own face, and second, to elucidate the *mechanism* by which IMS modulates the self-other distinction. The term IMS is used here to describe the situation whereby one individual experiences on her body and observes on someone else’s body the same sensory stimulation at the same body-part (e.g., face). Across all experiments, we introduced important methodological advances by using unfamiliar, gender-matched, faces and establishing a baseline of self-recognition performance prior to multisensory stimulation (c.f. [32], [33]). Using unfamiliar faces rules out the possibility that the bias in self-face recognition towards the other’s face could be accounted simply by face familiarity and affiliation with the other [32], while obtaining a baseline self-recognition prior to stimulation allows us to quantify the exact change in self-face recognition as a result of multisensory stimulation (cf. [33]). We investigated the interaction between self- and other-representations following IMS across four experiments that used psychophysical, psychophysiological and psychometric tasks. The first psychophysical task (Experiment 1) was designed to minimize the effect of cognitive expectations on the performance of the self-recognition task. The psychophysiological task (Experiment 2) was used to provide objective physiological evidence of the changes in the perception of the other’s face following synchronous IMS. The psychometric task (Experiment 3) was used to examine the changes in the subjective experience of the participants during synchronous and asynchronous IMS. Finally, the second psychophysical task (Experiment 4) was designed to determine the effect of IMS on self- and other-recognition separately. We hypothesized that a change in the categorical boundary between self and other, as a function of the recognition task (self or other) *and* the pattern of visuo-tactile stimulation, could reveal the extent to which the other is assimilated into the mental representation of the self or the reverse [16]. The novelty of the present work is that it provides with a methodology that allows evoking controlled changes in self-representations and quantifying these changes introspectively, behaviorally and physiologically. From a theoretical perspective, we propose a new account of self-recognition that goes beyond the classic mnemonic account by considering the role of online multisensory input for maintaining or updating the mental representation of one’s self.

## Experiment 1

### Materials and Methods

#### Participants

All experiments were approved by the Departmental Ethics Committees, Department of Psychology, Royal Holloway, University of London. All subjects in the four experiments reported here gave their informed consent to participate. Thirty-nine paid-participants (23 female; *M_age_* ± *SD* = 24±6) took part in Experiment 1.

#### Apparatus and materials

A digital photograph of the participants’ face with a neutral facial expression, taken prior to the experimental session, was converted to gray scale and mirror transposed [35]. A black template was used to remove non-facial attributes (e.g., background, hair, ears). Subsequently, a computerized morphing procedure was implemented (Abrasoft Fantamorph) to produce a sequence of photos in which the participant’s face merged with another person’s face in 1% morphing transitions. This sequence of photos had as end points the original photos of the participant’s face and the other person’s face. The 100 photos were saved as individual images.

In addition, a 120 s “induction movie” was produced to display the face of an unfamiliar individual, of the same gender as the participant, being touched on the right cheek with a cotton-bud at a frequency of approximately 0.5 Hz, each stroke covering a distance of approximately 2 cm from the zygomatic bone downwards. The movie would then be presented in full screen mode with a 20″ LCD-screen positioned 50 cm away from participants. A keyboard and Presentation® software were used to control stimuli delivery and collect participant’s responses.

Informed consent for recording videos and photographs and displaying them to other participants was obtained from all the participants that served as models for the stimuli in this and the other experiments reported here.

#### Procedure

First, participants performed a self-recognition task (pre-stimulation test). Participants saw a series of images, and for each of them they judged whether the face depicted “*looked more like their own face or more like the other person’s face”* using a two-alternative forced choice (2AFC) method. The images depicted a face with a varying degree of morphing between “self” and “other”.

A standard staircase procedure [36] was used to find the degree of morphing for which participants perceived the percentage of “self” and “other” in the morph to be the same (hereafter referred to as point of subjective equality or PSE). Two staircases differing in the degree of morphing used as a starting point (either a “100% self” or a “100% other”) and their direction (“self to other” or “other to self” direction, respectively) were randomly interleaved. In each trial of the task, the staircase, from which the morph was presented to participants, was randomly selected. We used a hybrid algorithm, in which two consecutive alike responses are required for a reversal when a change in response direction occurs [36]. The initial step size was 5% and reduced to 1% after the first reversal. Each staircase ended after four reversals, and the task ended after both staircases were completed. This task, in which participants were required to give judgments for single pictures, without being aware of the direction of change from one picture to the other, avoids potential errors of habituation and/or anticipation due to cognitive expectations [36].

PSE was calculated to reflect the degree of morphing for which participants were equally likely to judge the morph as “self” or as “other”. PSE values obtained for both interleaved staircases (“self to other” and “other to self” directions) were averaged for each experimental condition [37], [38]. We present this value as the maximum percentage of the “other” face contained in the PSE. For example, a PSE of 43% suggests that participants could not distinguish between self and other in the picture that contained 43% of the other-face and 57% of the self-face. Any increase in this value as a result of IMS would suggest an increase in the maximum percentage of the “other” face contained in the pictures judged as self.

**Figure 1:**
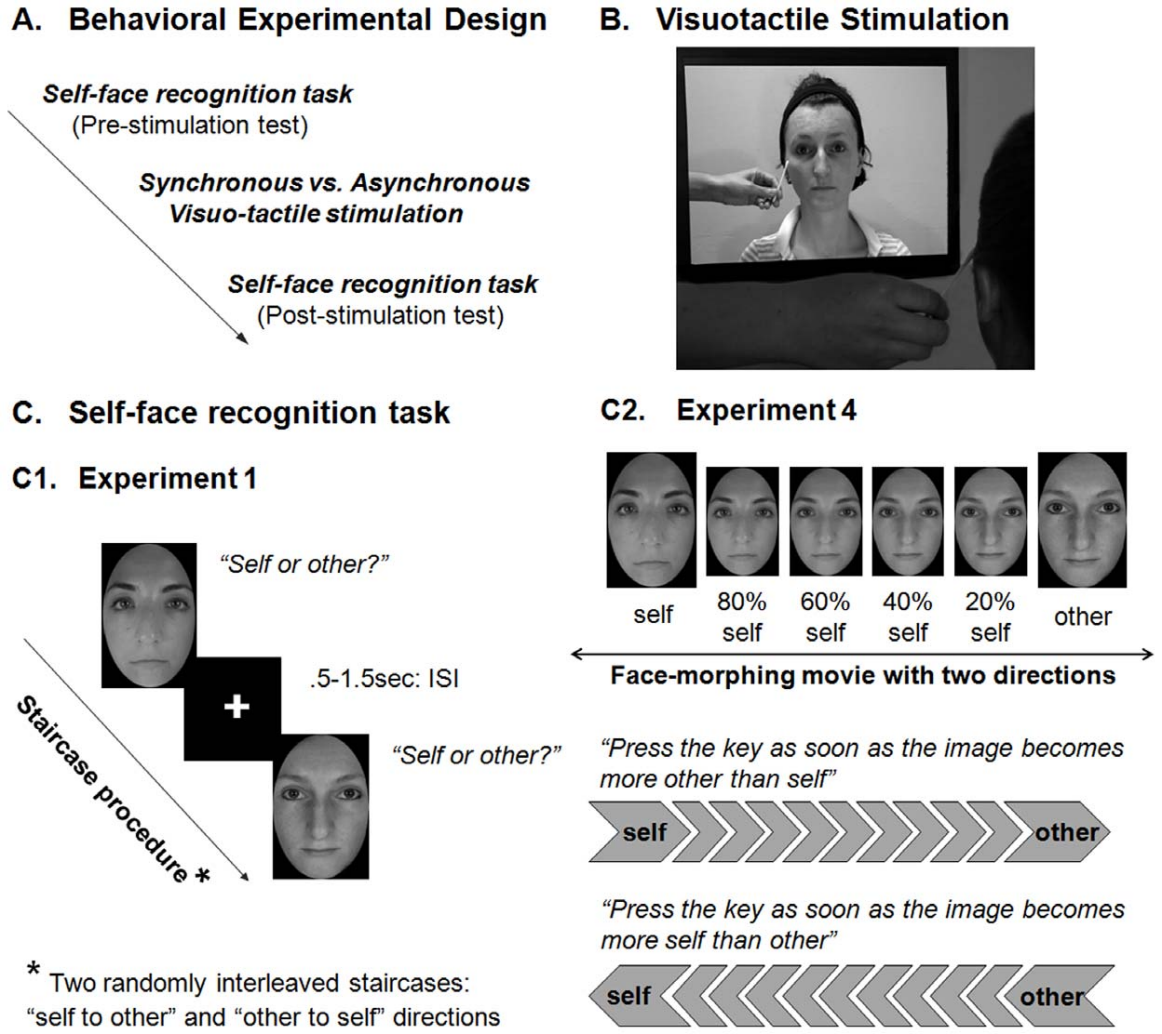
Experimental set-up during the visuo-tactile stimulation periods and behavioural experimental design. (A) Design of the experimental blocks, containing three phases: pre-stimulation test (*pre-test*), visuo-tactile stimulation and post-stimulation test (*post-test*). (B) Experimental set-up during the visuo-tactile stimulation period. (C) Behavioural task. Panel C1 shows the staircase procedure followed in Experiment 1, in which two staircases containing morphed images between self and other and differing in their direction of change, were randomly interleaved. Images were presented one after the other, with a random interstimulus interval (ISI) ranging between 0.5 and 1.5 s. For each image participants judged whether the face depicted looked *“more like their own face”* or *“more like the other person’s face”*. Panel C2 shows the morphing procedure, the direction of morphing (from “self to other” or from “other to self”) displayed in the two types of movies used, and the participants’ task in Experiment 4.

Upon completion of this baseline task, participants were exposed to the IMS phase. While the participant was looking at the other’s face being touched in the pre-recorded 120 s “induction movie”, the experimenter touched the participant’s face with an identical cotton bud on the specular congruent location (i.e., left side on the participant’s face, and right side on the other’s face; see Figure 1) either in synchrony, or asynchrony of 1 s, in different blocks. During stimulation, the experimenter listened to an audio file through headphones to pace the delivery of tactile stimulation. Next, to behaviorally quantify the effect of IMS on face recognition, participants performed (post-stimulation test) the same self-face recognition task as the one they had completed before the IMS phase.

Participants completed two blocks, one synchronous and one asynchronous, in two different sessions, separated by at least one day, their order counterbalanced across participants. Each experimental block contained three phases: pre-stimulation test (*pre-test*), visuo-tactile stimulation and post-stimulation test (*post-test*).

### Results and Discussion

**Figure 2:**
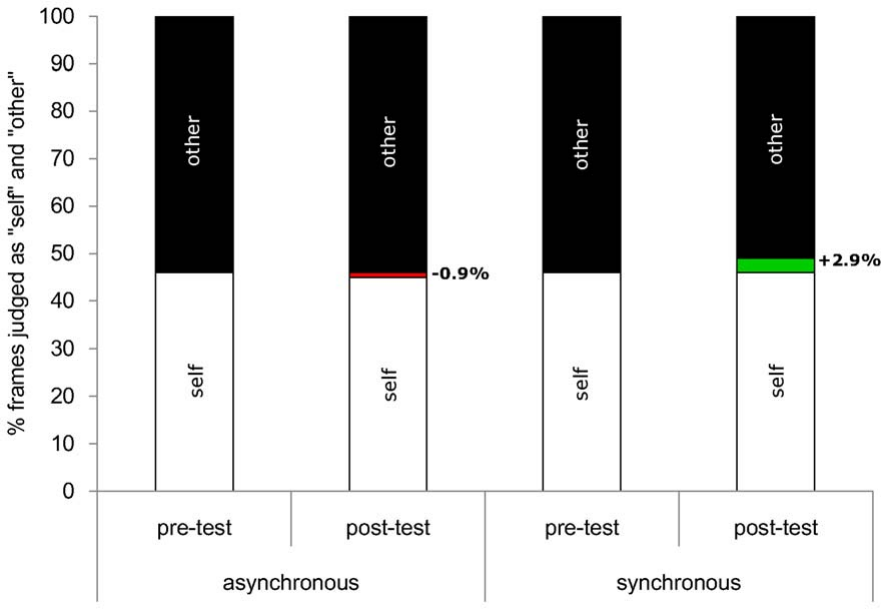
Results of Experiment 1. Mean percentage of frames perceived to look more like “self” or “other” as a result of the synchronous or asynchronous visuo-tactile stimulation and the timing of the test (pre-test vs. post-test). Positive changes (in green colour) indicate that the % of frames judged as “self” increased because participants accepted as “self-stimuli” morphed pictures that contained a larger % of the other’s face, relative to the pre-test. In contrast, negative changes (in red colour) indicate that the % of frames judged as “self” decreased because participants judged as self-stimuli morphed pictures that contained a smaller % of the other’s face, relative to the pre-test (0 = self, 100 = other).

The mean PSE values ± *SE* were, for the synchronous condition 45.91±*1.55* (pre-test) and 48.81±*1.71* (post-test), and for the asynchronous condition 46.22±*1.43* (pre-test) and 45.36±*1.67* (post-test). For all statistical tests alpha level was set at.05, 2-tailed, unless otherwise specified. A paired *t*-test comparison revealed no significant differences in the PSE prior to visuo-tactile stimulation across the synchronous and asynchronous conditions (*p* = .8), thus validating the choice of the pre-stimulation values as an appropriate baseline. PSE values were submitted in a 2x2 within-subjects ANOVA with type of visuo-tactile stimulation (i.e., synchronous vs. asynchronous) and timing of the test (i.e., pre-test vs. post-test) as factors. The two-way interaction between visuo-tactile stimulation and timing of the test was significant (*F*(1, 38) = 4.38, *p* = .043), while the main effects failed to reach significance (all *p*>.2). Planned paired *t*-test comparisons between pre- and post-tests showed that following synchronous (*t*(38) = 2.69, *p* = .011), but not asynchronous (*p* = .5), visuo-tactile stimulation, the PSE for self-recognition judgments significantly shifted towards the other’s face (see Figure 2). The change in the degree of morphing of PSE from pre- to post-stimulation reflects the change in self-face recognition following visuo-tactile stimulation. In other words, after synchronous stimulation pictures that contained a higher percentage of the other’s face (approximately 3%) were judged as self-images, as compared to the pre-test judgments. The significant interaction of visuo-tactile stimulation and timing of the test suggests that synchronous IMS produces changes in self-face representation relative to a baseline pre-stimulation performance, over and above the mere presence of multisensory stimulation (i.e., as compared to the asynchronous condition). These findings are compatible with the behavioral results of Tsakiris [32] and Sforza et al. [33], but importantly, they show, for the first time, that IMS between one’s own face and a completely unknown face can affect the mental representation of one’s own face.

Experiment 2 was designed to obtain objective physiological evidence of the changes in self-face representation following synchronous IMS. When people experience ownership over a foreign body, as a result of multisensory stimulation, they also exhibit increased physiological responses to threatening stimuli approaching the body that is attributed to the self [30]. We, therefore, investigated whether similar changes in physiological arousal can be observed for the synchronous IMS condition.

## Experiment 2

### Materials and Methods

#### Participants

The same group as in Experiment 1 took part, in a different session.

#### Apparatus and materials

The induction movies displaying an unfamiliar face lasted for either 40 s or 80 s. Previous research from similar bodily illusions (RHI) suggests that on average the illusion onset takes place approximately after 12 s of stimulation [39]. Towards the end of the movie, a knife appeared from the left side of the screen, moving towards the model’s face and being slightly inserted into the right corner of the model’s mouth. Apart from the asynchronous control condition, a second control condition was included to control for a general effect of seeing an object approaching the face. In this control condition, a non-threatening object (a spoon) approached, and made contact with, the other’s face at the same rate and through the same trajectory as the threatening object. This event, from the appearance of the object till the contact with the face, lasted 2 s. The spoon and the knife were similar in size, both with a black handle and being slightly tinted in red, either with fake blood or with red jam.

#### Procedure

Two sessions, synchronous and asynchronous, took place on different dates, separated by at least one day. For each session, four trials differing in the object appearing (knife vs. spoon) and the duration of the movie (40 s vs. 80 s) were presented in a counterbalanced order across participants, with the two spoon and two knife conditions always presented in pairs. To avoid anticipatory effects participants could not know in advance the length of the movie or the object that would appear on each trial.

To obtain objective physiological evidence of the changes in self-face representation following multisensory stimulation and in response to the presented threatening or non-threatening stimulus, we measured electrodermal activity (EDA) and heart rate (HR). EDA is a sensitive and valid indicator for the low arousal range, reflecting small variations in response to arousing stimuli [40], while heart rate deceleration (HRD) is a valid real-time measure for the higher arousal range and attention. An increase of attention is usually followed by a short term HRD [41]. For recording EDA, bipolar finger electrodes were attached to the first and second fingers, and a pulse transducer was attached to the thumb of the participants’ non-dominant hand. Physiological signals were sampled (at a rate of 1 kHz for HR, and 250 Hz for EDA signals) and amplified (AD Instruments).

The subjective experience of participants in response to the object approaching the other face was assessed with three statements presented in a random order at the end of each trial (see Table 1). Participants rated their level of agreement with the statements using a 7-item Likert scale. Participants also rated their emotional feelings using the 9-point valence and arousal pictorial scales of the Self-Assessment Manikin [42].

**Table 1:** Mean ratings (± *SD*) for each questionnaire item, and self-reported valence and arousal across conditions in Experiment 2.

	Threatening Object				Non-threatening Object			
Question	Sync	Async	*t*(38)	*p*	Sync	Async	*t*(38)	*p*
Q1. It seemed as if the knife/spoon was approaching my own face	−.18 *(1.3)*	−.99 *(1.6)*	3.69**	.001	−.36 *(1.4)*	−.81 *(1.3)*	1.97*	.056
Q2. It seemed as if the knife/spoon was touchingmy own face	−.59 *(1.4)*	−1.21 *(1.4)*	3.12**	.003	−.80 *(1.2)*	−1.19 *(1.3)*	1.86*	.070
Q3. It felt as if I experienced pain on myface when the knife/spoon touched the face	−.78 *(1.5)*	−1.34 *(1.4)*	2.86**	.007	−1.15 *(1.3)*	−1.5 *(1.3)*	1.85*	.073
Valence	4.15 *(1.8)*	4.64 *(1.8)*	−2.36**	.024	5.20 *(1.4)*	5.37 *(1.3)*	−.86	.397
Arousal	5.40 *(2.0)*	5.04 *(2.0)*	1.36	.18	4.44 *(1.8)*	4.38 *(1.7)*	.2	.843

* 1-tailed, **2-tailed

Higher values of valence and arousal mean that the emotional response to viewing the object was more positive and arousing.

### Results and Discussion

Based on previously reported studies [39], [43]–[45], we identified the intervals at which we expected a change in HR and EDA in response to the appearance of the object and its contact with the model’s face. We calculated change scores by comparing the activity in this region with that occurring during a baseline period before stimulus onset.

EDA and HR recordings were individually inspected for possible artifacts, which did not result in data exclusion. HRD was calculated for each trial by subtracting the heart interbeat interval (IBI) concurrent with the moment when the object touched the other’s face (IBI 0) from the third IBI preceding this point of contact (IBI -3) (baseline) [43]. EDA change scores were calculated for each trial by subtracting the mean response during 1 to 5 s following object onset from the mean response during the 1 s preceding object onset (baseline) [44]. This interval was chosen to be the region of interest, because changes in EDA are not as immediate as those in heart response, but they normally occur between 1 and 2 s after stimulus onset, although the response can be delayed up to 5 s [45]. EDA change scores were individually *z*-scored to control for variations in responsiveness [40], [46].

For all statistical tests alpha level was set at.05, 2-tailed, unless otherwise specified. Preliminary analyses did not show any difference in the baselines for HRD or EDA across the different trial conditions (*p*>.6), thus validating their choice. In addition, no difference was found across the different duration, 40 s and 80 s, conditions (*p* = .48), therefore we averaged the data from those conditions.

**Figure 3:**
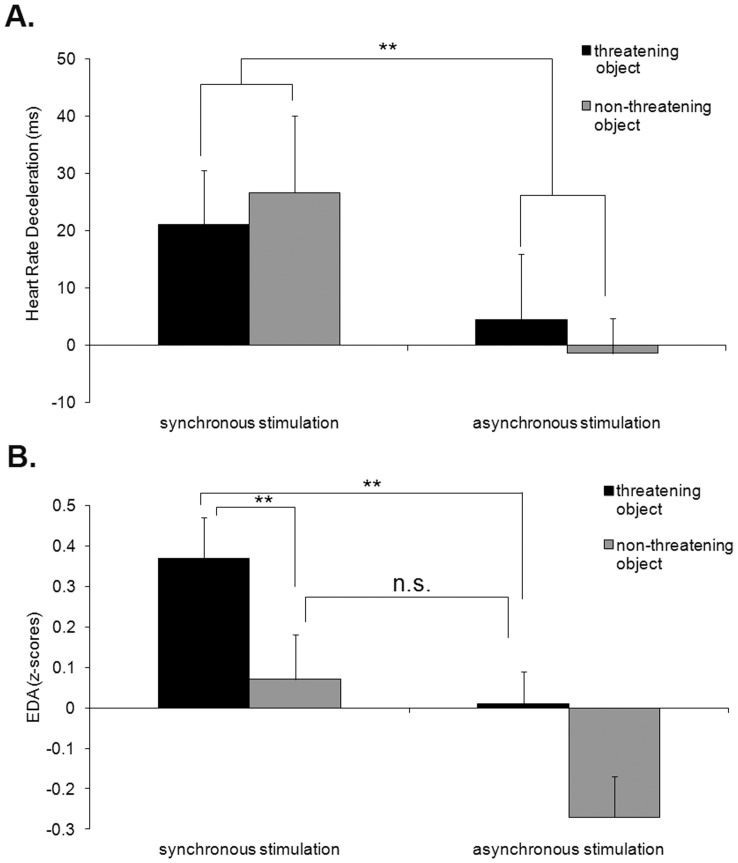
Results of Experiment 2. (A) Mean changes (± *SE*) in heart rate deceleration and (B) mean changes (± *SE*) in electrodermal activity (EDA) in response to the threatening and non-threatening object approaching the other’s face, following synchronous and asynchronous stimulation. ** denotes 2-tailed significant differences.

The mean HRD change scores ± *SE* in response to the different conditions relative to baseline were, following synchronous IMS 21.05±*9.5* (threatening object) and 26.65±*11.53* (non-threatening), following asynchronous IMS 4.44±*11.46* (threatening object) and −1.4±*6.02* (non-threatening). HRD scores were submitted in a 2x2 within-subjects ANOVA with type of visuo-tactile stimulation (i.e., synchronous vs. asynchronous) and object (i.e., knife vs. spoon) as factors. The main effect of visuo-tactile stimulation was significant (*F*(1,38) = 4.5, *p* = .04), while neither the main effect of the viewed object (*p* = .99) nor the interaction between factors were significant (*p* = .58, Figure 3*A*). The observed changes in HRD might reflect the general modulation of attention of visuo-tactile stimulation, independent of the kind of object that appeared, with synchronous IMS resulting in greater HRD.

The mean EDA change scores ± *SE* in response to the different conditions were, following synchronous IMS.37±.*1* (threatening object) and.07±.*09* (non-threatening), following asynchronous IMS.02±.*12* (threatening object) and −.27±.*12* (non-threatening). EDA change scores were submitted in a 2x2 within-subjects ANOVA with type of visuo-tactile stimulation (i.e., synchronous vs. asynchronous) and object (i.e., knife vs. spoon) as factors. Both the main effect of object (*F*(1,38) = 7.6, *p* = .009) and the main effect of visuo-tactile stimulation (*F*(1,38) = 6.4, *p* = .016) were significant (Figure 3B). The interaction of the two factors did not reach significance (*p* = .9), as it could be expected given the fact that the experience of seeing a knife is generally significantly more arousing than the experience of seeing a spoon (e.g., [47]). However, based on a large body of relevant findings [29], [30], [48] about the difference between the test condition (i.e., synchronous/threatening object) and either one of the two control conditions (i.e., synchronous/non-threatening and asynchronous/threatening), we predicted a priori that a significant difference would exist between our test and control conditions. We therefore used planned paired samples t-tests between the test and control conditions. The t-tests showed significantly greater EDA in response to the threatening object in the synchronous condition, than in either one of the two control conditions (“threatening object/asynchronous stimulation” (*t*(38) = 2.03, *p* = .049, 2-tailed), and “non-threatening object/synchronous stimulation” (*t*(38) = 2.24, *p* = .031, 2-tailed)). Differences in EDA between the two control conditions did not reach significance (*t*(38) = .30, *p* = .76).

The answers to the statements assessing the subjective experience of participants in response to the object approaching the other face during each condition were submitted in a 2×2 multivariate within-subjects ANOVA with type of visuo-tactile stimulation (i.e., synchronous vs. asynchronous) and object (i.e., knife vs. spoon) as factors, and the three statements (Q1–Q3) as dependent variables. Wilks’ Lambda was used as the multivariate criterion. Results revealed that the effect of visuo-tactile stimulation (*F*(3,36) = 3.17, *p* = .036; Λ = .791) and object (*F*(3,36) = 3.52, *p* = .025; Λ = .773) were significant, while the interaction was not (*p* = .282). The effect of visuo-tactile stimulation was significant (*p*<.05) for the three statements, revealing that after synchronous, as compared to asynchronous, stimulation participants had a greater feeling that the object was approaching (Q1), touching (Q2) and causing pain on (Q3) their own face. The effect of object was only significant for the last statement (*F*(1, 38) = 6.46, *p* = .015), which related to the experience of pain.

In addition, self-reported valence and arousal ratings revealed that there was a significant main effect of the object appearing on both valence and arousal scales (*F*(2,37) = 14.5, *p*<.001, Λ = .896), and a significant effect of the type of visuo-tactile stimulation on the valence scale (*F*(1,38) = 4.4, *p* = .043). The knife elicited more unpleasant and arousing emotional responses than the spoon, and the synchronous stimulation elicited a more unpleasant emotional response than the asynchronous stimulation (for a summary of introspective evidence see Table 1).

Experiment 3 was designed to investigate whether the changes in the subjective experience during synchronous IMS using a psychometric task, are consistent with the changes observed in the psychophysical task (Experiment 1) and other bodily illusions [24], [49].

## Experiment 3

### Materials and Methods

#### Participants

Twenty paid-participants (17 female; *M_age_* ± *SD* = 21±4) took part in Experiment 3.

#### Apparatus and materials

A similar apparatus as in Experiment 1, and similar induction movies, lasting for 120 s were used in Experiment 3.

#### Procedure

As in Experiments 1 and 2, while participants were looking at the other’s face being touched in the pre-recorded “induction movie”, the experimenter touched the participants’ face with an identical cotton bud on the specular congruent location either in synchrony, or asynchrony of 1 s. Two synchronous and two asynchronous trials were presented in randomized order across participants. The subjective experience of participants during each visuo-tactile condition was assessed with a questionnaire containing fourteen statements (adapted from [32], [49]), presented in a random order. Participants rated their level of agreement with the statements using a 7-item Likert scale.

### Results and Discussion

The mean ratings ± *SE* for each questionnaire item for the synchronous conditions are shown in Table 2. As it can be seen in Table 2, certain items did not result in affirmative ratings (Q3–6, Q10–12), while other items resulted in low yet affirmative values (Q1, Q2, Q7–9) following synchronous IMS. Our statistical analysis focused on the difference between the synchronous and asynchronous IMS to examine the effect of our manipulation on the Likert ratings for each questionnaire item.

**Table 2:** Mean ratings (± *SE*) for each questionnaire item in Experiment 3.

Question	Synchronous	Asynchronous	*z*	*p*
Q1. I felt the touch delivered in the other’s face	1.05 (.*4*)	−.55 (.*48*)	−2.2**	.028
Q2. The touch I felt was caused by the cotton bud touching the other’s face	.5 (.*41*)	−.95 (.*39*)	−3.1**	.002
Q3. The other’s face was my face	−.7 (.*42*)	−1.75 (.*35*)	−2.4**	.015
Q4. The other’s face was part of my body	−.65 (.*41*)	−2.0 (.*26*)	−2.9**	.004
Q5. The other’s face belonged to me	−1.25 *(.42)*	−1.75 (.*33*)	−1.6	.102
Q6. I was looking at my own reflection in a mirror rather than at the other’s face	−.6 *(.42)*	−1.95 (.*29*)	−2.8**	.005
Q7. The other’s face began to resemble my own face in terms of shape	.2 (.*49*)	−.55 (.*48*)	−2.4**	.016
Q8. The other’s face began to resemble my own face in terms of skin tone	.05 (.*45*)	−.95 (.*44*)	−2.2**	.025
Q9. The other’s face began to resemble my own face in terms of facial features	.3 (.*45*)	−.7 (.*44*)	−1.8*	.039
Q10. The other’s face would have moved if I had moved	−.05 (.*41*)	−.9 (.*41*)	−2.5**	.013
Q11. I was in control of the other’s face	−.7 (.*45*)	−1.7 (.*37*)	−2.7**	.007
Q12. My own face was out of my control	−.3 (.*45*)	−.95 (.*44*)	−1.6	.106
Q13. I couldn’t really remember how my face was	.6 (.*4*)	.1 (.*41*)	−1.3	.209
Q14. The experience of my face was less vivid than normal	.3 (.*42*)	−.25 (.*42*)	−1.2	.237

* 1-tailed, **2-tailed

**Figure 4:**
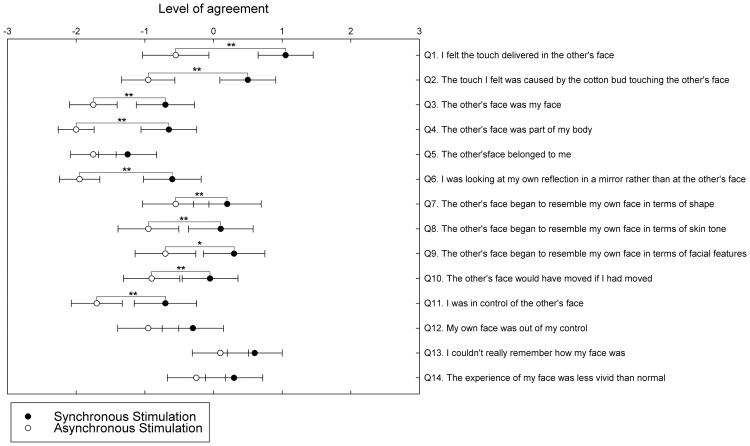
Results of Experiment 3. Mean ratings (± *SE*) for each questionnaire item across conditions. Participants rated their level of agreement with the statements using a 7-item Likert scale ranging from “strongly agree” (i.e., +3) to “strongly disagree” (i.e., −3). Significant differences between synchronous and asynchronous stimulation suggest changes in self-face representations as a result of the pattern of visuo-tactile stimulation. * denotes 1-tailed significant differences, and ** denotes 2-tailed significant differences.

For all statistical tests alpha level was set at.05, 2-tailed, unless otherwise specified. First, we tested whether the distributions of the obtained data were normal using the Shaphiro-Wilk test. None of the factors passed the normality test, therefore we used non-parametrical statistical tests to analyze the data (Wilcoxon Signed Ranks Test). Planned paired comparisons assessed the differences in the answers to each of the statements for the synchronous and asynchronous conditions. Synchronous, as opposed to asynchronous, IMS resulted in significant differences in subjective ratings across different dimensions (Figure 4 and Table 2), such as touch referral (Q1, Q2), identification with and ownership of the other’s face (Q3, Q4, Q6), changes in the perceived physical similarity between own and other face (Q7, Q8, Q9) and changes in the feelings of being able to move the other’s face and control over it (Q10, Q11). The significant differences between synchronous and asynchronous IMS are consistent with the pattern of the effects of multisensory stimulation in other bodily illusions, suggesting that, similarly with other body-parts, the experience of the face can be affected by multisensory input. However, the absence of affirmative ratings in the ownership and identification questions, while consistent with previous studies [33], [34], suggest that unlike other bodily illusions, synchronous IMS does not result in such strong sense of illusory ownership.

However, the questionnaire items that related to the source of the tactile sensation (Q1, Q2) and the perceived physical similarity between the other’s face and the self-face (Q7–9) resulted in affirmative ratings following synchronous IMS. The affirmative changes in items that addressed the physical similarity between self and other (see Q7–9) recall the phenomenology reported in bodily illusions [25]. Previous studies on the RHI have reported changes in the perceived similarity between felt and seen bodies whereby the rubber hand is perceived to become more similar to one’s own hand [24], [49]. While the introspective evidence of this experiment suggests a change in the perceived similarity of the other’s face relative to one’s own face, it remains unknown whether this effect is driven by a change in the recognition of one’s own face or the recognition of other’s face. This issue was investigated by Tsakiris [32] and Sforza et al. [33], who failed to find significant differences between self-recognition changes for the “self to other” and “other to self” directions [32] and between the judgments given under different instruction conditions (e.g., to evaluate the amount of “self” or to evaluate the amount of “other” contained in the morphs) [33]. However, we decided to revisit this issue given that our paradigm is novel in that the “other’s” face is unknown and unfamiliar. Experiment 1 did not allow for a proper investigation of an asymmetric effect for the two directions of morphing, given that the staircases with the two directions of morphing were not independent, as they were interleaved. Experiment 4 was, therefore, designed to specifically investigate the effect of visuo-tactile stimulation on self-recognition by quantifying the extent to which IMS affects self-face recognition or other-face recognition.

## Experiment 4

### Materials and Methods

#### Participants

Thirty volunteers (15 female; *M_age_* ± *SD* = 21±4) took part in Experiment 4.

#### Apparatus and materials

Similar materials as in Experiment 1 were used, except that in this case the induction movies lasted for 90 s, and the sequence of photos in which the participant’s face merged with the other’s face in 1% morphing transitions was used to produce two 100 s “morphing” movies, differing in their morphing direction. Thus the face on the movie was morphed either from 0% self to 100% self (“other to self” direction) or from 0% other to 100% other (“self to other” direction).

A similar set-up as the one described in Experiment 1 was employed, except that in this case E-prime software was used.

#### Procedure

Similar procedures as in Experiment 1 were followed except for the type of self-face recognition task performed before and after exposure to the multisensory stimulation, which in this case was adopted from Keenan et al. [35]. For this task, we used the face-morphing movies with two different morphing directions to assess the extent to which visuo-tactile stimulation results on the other being assimilated into the mental representation of the self or the reverse. For the “other to self” direction of morphing, participants stopped the movie with a key-press when they felt that the face looked more like self than other, and for the “self to other” direction of morphing, they stopped the movie when they felt that the face looked more like other than self. The same direction of morphing was used in the pre- and post-stimulation tests for each visuo-tactile condition.

Four experimental blocks, differing in the type of visuo-tactile stimulation (i.e., synchronous vs. asynchronous) and in the direction of morphing sequence (i.e., “other to self” vs. “self to other”), were completed, their order randomized across participants. If synchronous visuo-tactile stimulation affects both the representations of self- and other-face in the same way, we would expect similar changes in the post-stimulation test relative to the pre-stimulation test, independently of the direction of morphing. However, if, as observed in the studies on the RHI [49], synchronous visuo-tactile stimulation results only on the other being assimilated into the mental representation of the self, and not on the reverse, then we would expect specific changes in self-face recognition only for the “other to self” direction. We hypothesized that morphed pictures that contain more “other” than self will then be perceived as being more similar to the self-face and therefore participants will stop the movie earlier. On the contrary, changes in the “self to other” direction, would imply a change in other-face recognition, and would suggest that the self is perceived as being more similar to the other.

### Results and Discussion

The points at which participants stopped the movies were used to calculate the maximum percentage of the other face contained in the pictures that were judged as “self”. The mean percentages ± *SE* were, for the “other to self direction”, for the synchronous condition 50.1±*3.25* (pre-test) and 55.33±*3.0* (post-test), and for the asynchronous condition 52.83±*3.31* (pre-test) and 51.0±*3.24* (post-test); and for the “self to other direction”, for the synchronous condition 50.7±*3.2* (pre-test) and 47.83±*3.07* (post-test), and for the asynchronous condition 49.67±*3.51* (pre-test) and 49.43±*3.55* (post-test).

For all statistical tests alpha level was set at.05, 2-tailed, unless otherwise specified. We, first, investigated if there was a difference in the pre-stimulation self-recognition performance across the different conditions by submitting the mean pre-stimulation values in a 2×2 within-subjects ANOVA with the factors of visuo-tactile stimulation (i.e., synchronous vs. asynchronous) and direction of morphing sequence (i.e., “other to self” vs. “self to other”). No significant main effects or interaction were observed (all *p*>.4), thus validating the choice of the pre-stimulation values as an appropriate baseline. We, then, used a mixed ANOVA with the factors of type of visuo-tactile stimulation (i.e., synchronous vs. asynchronous), timing of the test (i.e., pre-test vs. post-test) and direction of morphing sequence (i.e., “other to self” vs. “self to other”) as within-subjects and gender as between-subjects.

**Figure 5:**
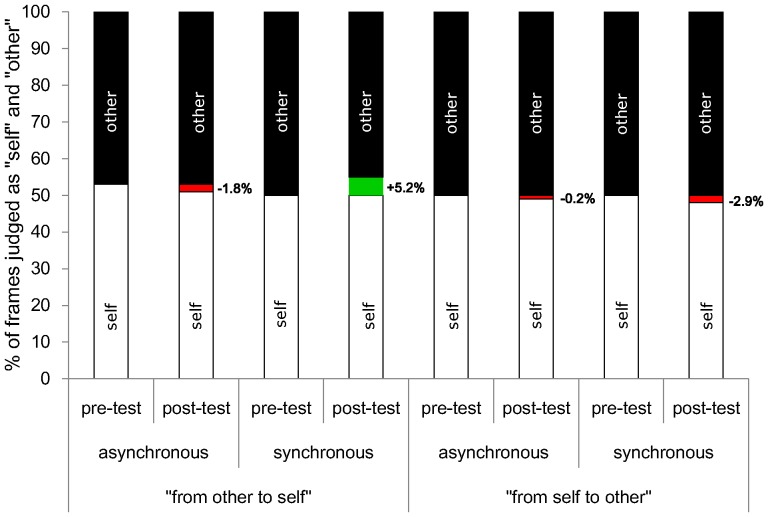
Results of Experiment 4. Mean percentage of frames perceived to look more like “self” or “other” as a result of the synchronous or asynchronous visuo-tactile stimulation, the timing of the test (pre-test vs. post-test) and the direction of morphing (“other to self” vs. “self to other”). Positive changes (in green colour) indicate that the % of frames judged as “self” increased because participants accepted as “self-stimuli” morphed pictures that contained a larger % of the other’s face, relative to the pre-test. In contrast, negative changes (in red colour) indicate that the % of frames judged as “self” decreased because participants judged as self-stimuli morphed pictures that contained a smaller % of the other’s face, relative to the pre-test (0 = self, 100 = other).

The 3-way interaction between type of stimulation, timing of test and direction of morphing was significant (*F*(1,29) = 4.3, *p* = .047), while the main effects and other interactions failed to reach significance (all *p*>.28; Figure 5). The significant interaction was driven by a specific effect of synchronous IMS on the “other to self” direction of morphing. Differences from pre- to post-test in the percentage of frames judged as “more self than other” between synchronous and asynchronous stimulation conditions were significant only when participants judged the stimuli in the morphing direction “other to self” (*t*(29) = 2.18, *p* = .037, 2-tailed), for which participants stopped the movie earlier (by approximately 5 seconds, corresponding to a 5% morphing difference) following synchronous stimulation. Therefore, on average participants accepted as self-stimulus a morphed picture that contained 55% of the other face. On the contrary, differences from pre- to post-test in the percentage of frames judged as “more self than other” between synchronous and asynchronous stimulation conditions when participants judged the stimuli in the morphing direction “self to other” did not reach significance (*t*(29) = .9, *p* = .375). The bias in self-face recognition as a result of synchronous IMS does not reflect a task-specific bias, because the pre-stimulation judgments were similar, independently of the morphing direction, or a general visual adaptation to the other’s face [50], because participants saw the other’s face for the same duration for both the synchronous and asynchronous visuo-tactile stimulation. Experiment 4 shows that synchronous visuo-tactile stimulation altered self-face representations, by producing changes in the recognition of the self-face, while recognition of the other’s face was not affected. These results might also indicate a specific change in the perceived similarity of the other face relative to self, but not the reverse, as discussed below.

In light of these findings, we revisited Experiment 1 in order to examine whether the asymmetric effect for the two directions of morphing observed in Experiment 4 was also present in Experiment 1, although in that experiment the directions of morphing were not independent, as the two staircases were interleaved. Thus, post-hoc paired t-test comparisons between synchronous and asynchronous conditions were conducted for each staircase separately. Differences in the percentage of frames judged as “more self” between synchronous and asynchronous stimulation were significant only when participants judged stimuli in the morphing direction “other to self” (*t*(38) = 2.17, *p* = .036, 2-tailed), while for the “self to other” morphing direction this difference did not reach significance (*t*(38) = 1.0, *p* = .3). As with Experiment 4, the significant changes in self-face recognition, observed only for the “other to self” staircase following synchronous stimulation, support the presence of an asymmetrical effect of interpersonal multisensory stimulation. However, this pattern should be interpreted with caution because the behavioral task of Experiment 1 was not designed to be sensitive to changes in the direction of morphing since the two staircases were interleaved.

### General Discussion

We developed an experimental situation that resembles the experience of looking at oneself into the mirror, albeit we replaced the “mirror reflection” of one’s face with another, unfamiliar, person’s face. Exposure to synchronous interpersonal multisensory stimulation (IMS), that is, synchronous vision and touch between one’s own face and the face of another unfamiliar person, evoked a measurable change in self-face recognition. This change was similar but subjectively not as strong as those changes observed in other body-illusions that use multisensory stimulation to manipulate body-awareness [24], [49]. The observed changes affected the categorical boundaries of self-other distinction (Experiments 1 and 4) as measured behaviorally. Interestingly, the change in the categorical boundaries of the two identities depended on the interaction between mode of stimulation and direction of morphing (Experiment 4). In relation to changes in the subjective experience (Experiment 3), we observed a significant difference between the synchronous and asynchronous IMS, in line with other bodily illusions, but unlike other bodily illusions, only few statements resulted in positive affirmative ratings. These were the ones that focused mainly on the perceived physical similarity between self and other, corroborating thus the behavioral findings. In terms of the autonomic arousal of the participants when viewing an object approaching the other’s face (Experiment 2), we observed a significant effect of synchronicity for both heart rate deceleration (HRD) and electrodermal activity (EDA), and a significant effect of the viewed object for EDA, while the interaction between the two factors did not reach significance. We discuss the observed effects and potential limitations and confounds in the following sections. We conclude by presenting a multisensory perspective on the construction of a mental representation of face identity.

### Changes in Autonomic Arousal

We sought to investigate the effect of synchronous IMS on the participants’ autonomic system. After synchronous IMS the participants’ autonomic responses such as HRD and EDA were increased when an object approached the other’s face. The observed larger HRD during synchronous, as compared to asynchronous, IMS, might reflect an increase in attention [41] towards the other’s face. It should be noted that a previous study which measured HRD in response to a person being threatened, observed from first- and third-person-perspective, did not find a significant difference in HRD to the threat occurring after synchronous as compared to the asynchronous touch condition, but they only found differences in HRD between the conditions when the person threatened was observed from first- as compared to third-person-perspective [51]. We here report a significant difference in HRD to a face being touched after the synchronous as compared to the asynchronous touch condition.

Second, for the EDA, we also observed a significant effect of synchronicity, such that synchronous IMS resulted in greater EDA responses relative to asynchronous stimulation, and independently of the viewed object, as evidenced by the lack of a significant interaction. Similarly, viewing a threatening object approaching the other’s face resulted in greater EDA responses than a non-threatening object, independently of the pattern of IMS. Taken together, the results suggest that EDA responses are modulated independently by the synchronicity of stimulation and the viewed object. The lack of a significant interaction cannot support the hypothesis that it was the induction of an enfacement illusion specifically that modulated arousal responses to threatening stimuli as a result of experiencing the other as oneself, rather than a purely attentional modulation. It could be argued that the observed effects simply reflect an attentional modulation that is specific to the synchronous IMS or a general increase in emotional arousal [40], [41]. For example, during synchronous IMS, a strong binding between one’s own tactile experiences and the visual impact on the other face results in increased attention to the other face, which can in turn explain the higher EDA responses as confirmed by the significant main effect of stimulation. This concern identifies a potential confound that requires an additional control condition with synchronous IMS but without changes in face representation. That would be analogous to the one used in the rubber hand illusion where, for example, synchronous multisensory stimulation is applied to a rubber hand that is placed in an incongruent posture with respect to the participant’s hand, a condition that does not elicit ownership [52]. Future studies should specifically address this point. In addition, we did not observe any significant differences in the EDA as a function of the duration of IMS (40 s versus 80 s). However, the exact time-onset of the enfacement illusion remains unknown, and future studies should address this issue.

Based on a priori hypotheses derived from previous studies on other bodily illusions [30], [39], we also investigated the extent to which our test condition (i.e., synchronous IMS with threatening object approaching) was significantly different from either control condition and the results revealed some preliminary supportive findings. Thus, EDA was significantly higher in response to a threat towards the other’s face following synchronous, as compared to asynchronous, IMS. EDA was also higher in the test condition compared to a non-threatening object, approaching the other’s face following synchronous IMS, which shows that the increase in arousal is threat-related and not only due to the general effect of seeing an object approaching the face after synchronous IMS. The observed changes in arousal for the critical test condition were consistent with those reported in comparable studies on bodily illusions (e.g., [30], [39]). When people experience ownership over a foreign body, as a result of multisensory stimulation, they also exhibit increased arousal responses to threatening stimuli approaching this newly owned body [30]. Therefore, despite the lack of a significant interaction, these planned contrasts provide some tentative support for an effect of synchronous IMS on autonomic reactivity related to stimuli, and perhaps more so for threatening stimuli, approaching the other’s face.

### Changes in Subjective Experience

The experimental manipulation of the synchrony or asynchrony of IMS produced significant changes in the participants’ subjective ratings. Following synchronous IMS, participants accepted statements referring to the source of tactile sensation (Q1, Q2) and the change in resemblance between the other’s face and their own face (Q7, Q8, Q9), while they denied the same statements following asynchronous IMS. In addition, while certain statements resulted in negative ratings following both synchronous and asynchronous IMS (Q3, Q4 and Q6), the ratings between the synchronous and asynchronous conditions were significantly different, suggesting that participants showed less negation following synchronous IMS for these statements (e.g., “looking at one’s mirror reflection, rather than at someone else”). Overall, this pattern is consistent with the reported changes in subjective experience in other studies reporting the influence of multisensory stimulation in face recognition [33], [34], but it should be noted that in our study the mean value for the critical statement Q3 (“I felt as if the other’s face was my face”) is numerically higher than the ones reported in previous studies [33], [34]. The pattern of results is also consistent with that reported for other bodily illusions [49], although it seems that other bodily illusions (e.g., RHI) produce stronger phenomenological effects, as reported by participants. Synchronous, as compared to asynchronous, IMS resulted in significant differences in participants’ ratings of their experience, but unlike other bodily illusions, the evidence for strong and positive changes in subjective experience was limited to a change in “touch referral” and the perceived physical similarity between the two faces. Of interest, this pattern shows that looking at someone else’s face being touched in synchrony resulted in a positive change in the experience of the source of sensation, that is, a referral of the felt touch on the vision of touch delivered on the other’s face (see Q1, Q2). These items are important as they reflect the subjective experience of a key process of “touch referral” that has been implicated in the inducement of similar bodily illusions [53]. In addition, the overall affirmative ratings in questions relating to the perceived similarity of the other’s face (see Q7–9) following synchronous IMS point to a key change in subjective experience and are consistent with the behavioral pattern as discussed below.

### Behavioral Changes in Self-recognition

Experiment 1 was designed to control for potential confounds reported in previous studies, such as the use of a familiar face [32], [33] and the lack of a pre-test baseline self-recognition performance [33]. By using a staircase procedure, which consisted of two randomly interleaved staircases moving from one end point (e.g., “self-face”) to the other (e.g., “other-face”), we showed that synchronous IMS changed self-other recognition performance, by approximately 3%, relative to both a baseline pre-test measure and asynchronous IMS, even when participants are exposed to an unfamiliar face during IMS. The percentage of change reported in Experiment 1 is comparable to that reported in similar studies that used a familiar “other” face (1.8% in [32], and 4.4% in [33]).

Could the observed differences between synchronous and asynchronous IMS reflect differences in familiarization with the model’s face? This seems unlikely, because across conditions, participants were exposed to the model’s face for equal duration. Could the observed differences reflect a task-related bias? First, the fact that differences were specific to synchronous IMS suggests that this is unlikely. Second, previous studies (e.g., [50]) used an identification or classification task to determine the perceived categorical boundary between two facial identities in a morphed continuum, and found that the boundary position for faces familiar to the observer does not significantly differ from the physical 50% morph. Interestingly, for unfamiliar faces, as was the case in our experiments, the boundary shifts towards the most distinctive end-point (i.e., the self-face). Here, we used an unfamiliar face, and synchronous IMS seems to reverse this pattern by shifting categorical boundaries towards the unfamiliar face, suggesting that a higher percentage of the other face is assimilated in the mental representation of the self-face. This is contrary to what would have been predicted by shift of the boundary to the most distinctive end-point.

Experiment 4 used a behavioral task to differentiate between changes in recognition of the self, relative to the other’s face, and changes in recognition of other face, relative to the self-face [16], [35]. Synchronous IMS specifically affected recognition of the self-face, as statistically significant changes were observed only for the direction of morphing that presented a transition from other to self. When participants saw the face of the other being slowly morphed into the self-face, and were asked to indicate when the face looks more like self, they stopped the movie significantly earlier compared to the pre-stimulation test. This pattern suggests that, following IMS, participants accepted as self-stimuli morphed faces that contained 5% more of the other’s face. Importantly, no similar effects were observed for the reverse direction of morphing (i.e., “self to other”). This asymmetric effect for the two directions of morphing could also be observed in Experiment 1, but was not found in previous studies where the other’s face was a familiar one [32], [33]. Previous studies failed to find significant differences between self-recognition changes for the “self to other” and “other to self” directions in a video morphing from one face to the other [32], a task identical to the one used in our Experiment 4, and between the judgments of morphed images under different instruction conditions (either to evaluate the amount of “self” or to evaluate the amount of “other” contained in the morphs) [33], a task related to the one in our Experiment 1. Importantly, the main difference between previous studies and ours is that in our paradigm the “other’s” face was completely unfamiliar to participants, and therefore it is possible that the lack of directional effect in past studies was confounded by the high familiarity of the model’s face. The presence of an asymmetric effect here is also consistent with the effect of neural interference by means of rTMS over the rTPJ that has been shown to affect recognition performance when the morph moved from other to self, but not the reverse [16]. Heinisch et al. [16] argued that disrupting neural processing in rTPJ makes self-recognition performance less conservative (i.e., increasing the likelihood of accepting other faces as one’s own face), while other-face recognition is unaffected (i.e., the likelihood of judging one’s own face as that of someone else is not changing). Consistent with this pattern, our results show that morphed instances of the other’s face are perceived as self-stimuli, whereas morphed instances of the self-face are not perceived as other-stimuli.

Is it possible that synchronous IMS disrupts face recognition performance in general? This seems unlikely, given that Tsakiris [32] showed the behavioral effect to be specific to the face seen during visuo-tactile stimulation, and not to other familiar faces that were not seen during stimulation. In addition, the fact that in Experiment 4, no changes were observed between pre-test and post-test in the “self to other” direction of morphing following synchronous IMS suggests that the effect of IMS is restricted to recognition of one’s own face.

We, therefore, show that synchronous IMS between one’s own face and that of another unknown individual can change the categorical boundary between self-other (Experiment 1 and 4). Moreover, this change depends on the interaction of the pattern of stimulation and the direction of morphing (Experiment 4), that makes the other’s face to be perceived as self-face. In principle, categorical boundaries should not be affected by the direction of morphing alone. For example, it has been found that when the two end-points of a continuum are the self-face and an unknown face, the categorical boundaries are dependent on the perceived face similarity between self and other but not on the direction of morphing [13], [54]–[56]. Aside from the rTMS study over the rTPJ that showed an asymmetric effect on categorical boundaries depending also on the direction of morphing [16], we show that synchronous IMS can also elicit an asymmetric effect, such that the other’s face is perceived as self-face, while the reverse did not occur. We propose that this change is the result of a change in the perceived similarity of the other’s face relative to the mental representation of the self-face. Given the previously reported effects of perceived similarity on categorical boundaries and the present findings that synchronous IMS affects the perceived similarity of the other’s face relative to the self-face (see Experiment 3, Q7 to Q9, and Experiment 4), we suggest that synchronous IMS produces a quantifiable change in self-recognition, such that the “other” becomes part of the mental representation of one’s own face. Pictures that contained more frames of the other were perceived as more similar to the self. This result might be interpreted as the “other” becoming more similar to the self due to the effect of synchronous visuo-tactile stimulation. This is also consistent with the previously reported direction of changes in the representation of one’s hand and a rubber hand following multisensory stimulation. Longo et al. [24] reported that participants who experienced the RHI also felt that the rubber hand was becoming more similar to their own hand, but not the reverse. Similarly, changes in self-face representation are caused by changed perceptions of the other’s face, rather than by changed perceptions of one’s own face.

This effect might also depend on or impact upon processes implicated in social cognition. It has been suggested that the perceived similarity of other people to one’s self is the starting point for inferring that others have similar psychological processes, including perceptions and emotions, as one’s self (see the “like me” process [57]). Our results provide further support to these theories because following synchronous IMS, the “other” is perceived to be more “like me”. Perceived similarity between self and other might also impact upon social cognition processes. For example, Paladino et al. [34], using an experimental paradigm similar to ours, showed that IMS altered the social perception of participants towards the other person: following synchronous vs. asynchronous IMS, participants reported a higher self-other merging measured in terms of inner states, closeness and physical attraction, and they tended to conform more with the other.

### A Multisensory Perspective on Self-representations

Overall, synchronous IMS resulted in a quantifiable and unidirectional change in the mental representations of one’s face, as measured behaviorally. Evidence for changes in autonomic responses and in the subjective experience of self-identification were broadly consistent with patterns observed in other bodily illusions, but they were not as potent. Importantly, one consistent pattern that emerged from both the behavioral and the introspective evidence was that shared multisensory experiences between self and other can change the perceived physical similarity of others relative to one’s self. This effect of multisensory input has interesting theoretical implications for our understanding of the plasticity of self-representations in relation to both identity and self-other boundaries.

Previous studies on self-face recognition and sense of identity have focused on visual processing and the role of mnemonic representations of one’s appearance. The present investigation goes beyond this classic mnemonic account of self-face representations by highlighting a previously unexplored connection between basic processes of multisensory integration and the plasticity of self-identity. Representations of self-identity must possess sufficient plasticity to ensure both the assimilation of changes and a sense of continuity over time. Such processes of adaptive reorganization of self-representations allow the narrative “I” to experience the same self as yesterday and “the feeling of the same old body always there” [58], even though one’s self and body are changing. Multisensory integration provides a plausible mechanism for constructing a self-representation, and for the subsequent assimilation of changes and updating of self-representations. In fact, it would be difficult to understand how infants are capable of recognizing their mirror reflection and forming a mental self-representation from their mirror-reflection, unless they can first integrate somatosensory signals with visual feedback [6]. Our experiments show how mental representations of our physical appearance are modulated by current online multisensory input by means of a change in the perceived similarity between an external stimulus (i.e., the other’s face or one’s mirror reflection) and the mental representation of an internal stimulus (i.e., one’s own face). This change in perceived similarity is caused by the synchrony of multisensory stimuli, which in the context of body-awareness has been shown to determine whether external stimuli can be experienced as part of the self or not [25]. This functional account of the interaction between multisensory input and self-representations is grounded on the known functional engagement of frontoparietal areas in the right hemisphere.

Uddin et al. [59] suggest that there are at least two neural networks involved in representing self and others. The frontoparietal mirror neuron system (MNS), which is involved in processing the physical self [11], and a network composed of the cortical midline structures (CMS) including the medial prefrontal cortex, the anterior cingulate cortex and the precuneus which is involved in the more abstract, evaluative processing of self and others. Interestingly, the MNS network, the insula and the TPJ are often engaged in the processing of body movements of self and others, as well as during multisensory perception and integration. For example, Ishida et al. [60] showed that visuo-tactile neurons in the parietal cortex display mirroring properties and can be used to link self and other body representations. Insula activations in the right hemisphere have been reported during bodily illusions of body-ownership caused by multisensory integration [61], as well as during self-face recognition [12], and the mapping of observed bodily states on one’s own body [62]. Finally, the right TPJ has been shown to engage in the filtering of multisensory percepts that may be assigned to one’s own body or not [63] and in the maintenance of a 1^st^ person perspective [64]. These results suggest that self-other distinction and recognition of the physical self might be based upon specific processes of multisensory perception.

Interestingly, the same neural structures that represent the sentient self may also be used in social interactions. For example empathetic responses [34], [62] may be based on mapping the others’ bodily states to the representation of the one’s own bodily states. This mapping may also depend on the perceived physical similarity between self and other [65]. Our results support a model of self-awareness according to which our sense of self is plastically affected by multisensory information as it becomes available during self-other interactions. This model provides a functional explanation of how the “I” comes to be identified with “me”, allowing this “me” to be represented as an object for the others, but also for one’s own self.

## References

[1] Gallup GGJ (1970). Chimpanzees: Self-Recognition.. Science.

[2] Rochat P, Zahavi D (2011). The uncanny mirror: A re-framing of mirror self-experience.. Consciousness & Cognition.

[3] de Waal FB, Dindo M, Freeman CA, Hall MJ (2005). The monkey in the mirror: Hardly a stranger.. Proceedings of the National Academy of Sciences of the United States of America.

[4] Bertenthal BI, Fischer KW (1978). Development of self-recognition in the infant.. Child Development.

[5] Zahavi D, Roepstorff A (2011). Faces and ascriptions: mapping measures of the self.. Conscious & Cognition.

[6] Povinelli DJ, Simon BB (1998). Young children's understanding of briefly versus extremely delayed images of self: Emergence of the autobiographical stance.. Developmental Psychology.

[7] Lewis M (2006). The emergence of consciousness and its role in human development.. Annals of the New York Academy of Sciences.

[8] Gillihan SJ, Farah MJ (2005). Is self special? A critical review of evidence from experimental psychology and cognitive neuroscience.. Psychological Bulletin.

[9] Feinberg TE, Keenan JP (2005). Where in the brain is the self?. Consciousness and Cognition.

[10] Devue C, Brédart S (2011). The neural correlates of visual self-recognition.. Consciousness & Cognition.

[11] Uddin LQ, Kaplan JT, Molnar-Szakacs I, Zaidel E, Iacoboni M (2004). Self-face recognition activates a frontoparietal “mirror” network in the right hemisphere: an event-related fMRI study.. NeuroImage.

[12] Devue C, Collette F, Balteau E, Degueldre C, Luxen A (2007). Here I am: The cortical correlates of visual self- recognition.. Brain Research.

[13] Kircher TTJ, Senior C, Phillips ML, Rabe-Hesketh S, Benson PJ (2001). Recognizing one’s own face.. Cognition.

[14] Uddin LQ, Rayman J, Zaidel E (2005). Split-brain reveals separate but equal self-recognition in the two cerebral hemispheres.. Consciousness & Cognition.

[15] Uddin LQ, Molnar-Szakacs I, Zaidel E, Iacoboni M (2006). rTMS to the right inferior parietal lobule disrupts self-other discrimination.. Social Cognitive and Affective Neuroscience.

[16] Heinisch C, Dinse HR, Tegenthoff M, Juckel G, Brune M (2010). An rTMS study into self-face recognition using video-morphing technique.. Social Cognitive and Affective Neuroscience.

[17] Brady N, Campbell M, Flaherty M (2004). My left brain and me: A dissociation in the perception of self and others.. Neuropsychologia.

[18] Brady N, Campbell M, Flaherty M (2005). Perceptual asymmetries are preserved in memory for highly familiar faces of self and friend.. Brain & Cognition 58, 334–342.

[19] Brédart S (2003). Recognising the usual orientation of one's own face: The role of asymmetrically located details.. Perception.

[20] Keenan JP, Wheeler MA, Gallup GG, Pascual-Leone A (2000). Self-recognition and the right prefrontal cortex.. Trends in Cognitive Sciences.

[21] Tong F, Nakayama K (1999). Robust representations for faces: Evidence from visual search.. Journal of Experimental Psychology: Human Perception and Performance.

[22] Rochat P (2003). Five levels of self-awareness as they unfold early in life.. Consciousness & Cognition.

[23] Tsakiris M (2010). My body in the brain: A neurocognitive model of body-ownership.. Neuropsychologia.

[24] Longo MR, Schüür F, Kammers MPM, Tsakiris M, Haggard P (2009). Self awareness and the body image.. Acta Psychologica.

[25] Botvinick M, Cohen J (1998). Rubber hands ‘feel’ touch that eyes see.. Nature.

[26] Ehrsson HH (2007). The experimental induction of out-of-body experiences.. Science.

[27] Lenggenhager B, Tadi T, Metzinger T, Blanke O (2007). Video ergo sum: Manipulating bodily self-consciousness.. Science.

[28] Petkova VI, Björnsdotter M, Gentile G, Jonsson T, Li TQ (2011). From part- to whole-body ownership in the multisensory brain.. Current Biology.

[29] Petkova VI, Khoshnevis M, Ehrsson HH (2011). The perspective matters! Multisensory integration in ego-centric reference frames determines full body ownership.. Frontiers in Psychology.

[30] Petkova VI, Ehrsson HH (2008). If I were you: Perceptual illusion of body swapping.. PLoS ONE.

[31] Blanke O, Metzinger T (2009). Full-body illusions and minimal phenomenal selfhood.. Trends in Cognitive Sciences.

[32] Tsakiris M (2008). Looking for myself: Current multisensory input alters self-face recognition.. PLoS One.

[33] Sforza A, Bufalari I, Haggard P, Aglioti SM (2010). My face in yours: Visuo-tactile facial stimulation influences sense of identity.. Social Neuroscience.

[34] Paladino M-P, Mazzurega M, Pavani F, Schubert TW (2010). Synchronous multisensory stimulation blurs self-other boundaries.. Psychological Science.

[35] Keenan JP, McCutcheon B, Freund S, Gallup GG, Sanders G (1999). Left hand advantage in a self-face recognition task.. Neuropsychologia.

[36] Meese TS (1995). Using the standard staircase to measure the point of subjective equality: A guide based on computer simulations.. Perception & Psychophysics.

[37] Watson TL, Clifford CWG (2003). Pulling faces: An investigation of the face-distortion aftereffect.. Perception.

[38] Webster MA, Kaping D, Mizokami Y, Duhamel P (2004). Adaptation to natural facial categories.. Nature.

[39] Ehrsson HH, Spence C, Passingham RE (2004). That's my hand! Activity in premotor cortex reflects feeling of ownership of a limb.. Science.

[40] Boucsein W (1992). Electrodermal activity.. New York: Plenum Press.

[41] Lang PJ, Bradley MM, Cuthbert BN (1990). Emotion attention and the startle reflex.. Psychological Review.

[42] Lang PJ, Sidowski JB, Johnson JH, Williams TA (1980). Behavioral treatment and bio-behavioral assessment: computer applications..

[43] Moor BG, Crone EA, van der Molen MW (2010). The heartbrake of social rejection: Heart rate deceleration in response to unexpected peer rejection.. Psychological Science.

[44] Dimberg U (1990). Facial electromyography and emotional reactions.. Psychophysiology.

[45] Edelberg R, Brown CC (1967). Electrical properties of the skin..

[46] Venables PH, Christie MJ, Prokasy WF, Raskin DC (1973). Mechanisms instrumentation recording techniques and quantification of responses..

[47] Lang PJ, Bradley MM, Cuthbert BN (2008). International affective picture system (IAPS): Affective ratings of pictures and instruction manual. Technical Report A-8.. Gainesville, FL: University of Florida.

[48] Petkova VI, Ehrsson HH (2009). When right feels left: Referral of touch and ownership between the hands.. PLoS ONE.

[49] Longo MR, Schüür F, Kammers MPM, Tsakiris M, Haggard P (2008). What is embodiment? A psychometric approach.. Cognition.

[50] Leopold DA, Rhodes G, Müller K-M, Jeffery L (2005). The dynamics of visual adaptation to faces.. Proceedings of the Royal Society Series B: Biological Sciences.

[51] Slater M, Spanlang B, Sanchez-Vives MV, Blanke O (2010). First person experience of body transfer in virtual reality.. PLoS ONE.

[52] Guterstam A, Petkova VI, Ehrsson HH (2011). The illusion of owning a third arm.. PLoS ONE.

[53] Makin TR, Holmes NP, Ehrsson HH (2008). On the other hand: Dummy hands and peripersonal space.. Behavioural Brain Research.

[54] Angeli A, Davidoff J, Valentine T (2008). Face familiarity distinctiveness and categorical perception.. The Quarterly Journal of Experimental Psychology.

[55] Tanaka J, Giles M, Kremen S, Simon V (1998). Mapping attractor fields in face space: the atypicality bias in face recognition.. Cognition.

[56] Yoon HW, Kircher TT (2005). The influence of face similarity in the case of the perception of morphed self-face International.. Journal of Neuroscience.

[57] Meltzoff AN (2007). 'Like me': A foundation for social cognition.. Developmental Science.

[58] James W (1890). The principles of psychology.. New York: H. Holt and Company.

[59] Uddin LQ, Iacoboni M, Lange C, Keenan JP (2007). The self and social cognition: the role cortical midline structures and mirror neurons.. Trends in Cognitive Sciences.

[60] Ishida H, Nakajima K, Inase M, Murata A (2010). Shared mapping of own and others' bodies in visuotactile bimodal area of monkey parietal cortex.. Journal of Cognitive Neuroscience.

[61] Tsakiris M, Hesse MD, Boy C, Haggard P, Fink GR (2007). Neural signatures of body ownership: a sensory network for bodily self-consciousness.. Cerebral Cortex.

[62] Singer T, Seymour B, O'Doherty J, Kaube H, Dolan RJ (2004). Empathy for pain involves the affective but not sensory components of pain.. Science.

[63] Tsakiris M, Costantini M, Haggard P (2008). The role of the right temporo-parietal junction in maintaining a coherent sense of one's body.. Neuropsychologia.

[64] Ionta S, Heydrich L, Lenggenhager B, Mouthon M, Fornari E (2011). Multisensory mechanisms in temporo-parietal cortex support self-location and first-person perspective.. Neuron.

[65] Avenanti A, Sirigu A, Aglioti SM (2010). Racial bias reduces empathic sensorimotor resonance with other-race pain.. Current Biology.

